# Comparison between the effect of adding microhydroxyapatite and chitosan on surface roughness and Microhardness of resin modified and conventional glass ionomer cements

**DOI:** 10.4317/jced.55996

**Published:** 2021-08-01

**Authors:** Farahnaz Sharafeddin, Zahra Jowkar, Somaye Bahrani

**Affiliations:** 1 Professor, Biomaterials Research Center, Department of Operative Dentistry, School of Dentistry, Shiraz University of Medical Sciences, Shiraz, Iran; 2 Assistant professor, Oral and Dental Disease Research Center, Department of Operative Dentistry, School of Dentistry, Shiraz University of Medical Sciences, Shiraz, Iran; 3 Postgraduate Student, Department of Operative Dentistry, School of Dentistry, Shiraz University of Medical Sciences, Shiraz, Iran

## Abstract

**Background:**

This study aimed to compare the effect of chitosan (CH) and hydroxyapatite (HP) on the surface roughness and microhardness of a conventional glass ionomer cement (CGIC) and a resin modified glass ionomer cement (RMGIC).

**Material and Methods:**

60 disk-shaped specimens (2mm x 6mm) were prepared in 6 groups; group I: CGIC, group II: RMGIC, group III: CGIC + 15% volume CH solution in liquid, group IV: CGIC +10% weight micro-HP in powder, group V: RMGIC + 15% volume CH, group VI: RMGIC + 10% weight micro-HP. After storage in deionized water at room temperature for 24 hours, the surface roughness and microhardness of the specimens were measured using a surface profilometer and Vickers microhardness (VHN) tester, respectively. Data were analyzed using two-way ANOVA, Tukey HSD test and paired t-test (*P*<0.05).

**Results:**

The microhardness values of RMGIC and CGIC decreased significantly with the addition of micro-HP (*P*<0.001). None of the CH-containing GICs showed significant changes in microhardness (*P* = 0.552). The VHN values of CGIC were higher than RMGIC, regardless of the added substance (*P*<0.001). The surface roughness (Ra) values (μm) of both RMGIC and CGIC decreased significantly with the addition of CH (*P* = 0.004). The incorporation of micro-HP into GICs did not have a significant effect on surface roughness values (*P* = 0.700). The RMGIC showed less Ra values compared to the CGIC regardless of the added substance (*P*<0.001). The lowest and highest Ra values were observed in RMGIC + CH and CGIC + micro-HP groups, respectively.

**Conclusions:**

The addition of CH to GIC and RMGIC reduced the surface roughness and did not have an adverse effect on the microhardness. Mixing GIC and RMGIC with micro-HP resulted in microhardness reduction and did not affect the surface roughness.

** Key words:**Glass ionomer, hydroxyapatite, chitosan, hardness, surface roughness

## Introduction

The development of restorative materials in the modern era of preventive and conservative dentistry should be towards producing bioactive materials which provide therapeutic effects. Glass ionomer cement (GIC) is one of the most common biomaterials used in dentistry. Due to its favorable properties such as biocompatibility, anti-cariogenicity, antibacterial effects, the ability to adhere to enamel and dentin without the need of an adhesive, and a low thermal expansion coefficient similar to tooth structure, GIC is widely used as a restorative material, sealant, luting cement and cavity base (1). However, some disadvantages such as early moisture sensitivity, brittleness, low mechanical strength, and low resistance to wear and surface roughness limit the clinical applications of this material (2).

Numerous modifications have been made in GICs to improve their physical, mechanical and biological properties. Resin modified glass ionomer cements (RMGICs) were introduced to improve the mechanical properties and reduce GIC sensitivity to moisture and dehydration during the initial setting (3,4). The addition of different fillers such as silver nanoparticles, titanium dioxide nanoparticles, and polyethylene fiber into GICs has been previously evaluated (5-7). The addition of the fillers into GICs was shown to reinforce GICs and improved GICs’ mechanical properties (3,5,6).

Hydroxyapatite (HP), which is the main mineral component of the teeth and bone structures, is a calcium and phosphorus-containing bioceramic. Efforts have been made to combine HP particles with GICs (3). Studies have shown that HP improves the fluoride release as well as the mechanical properties of GICs such as flexural strength and compressive strength (1). Chitosan (CH) is also a linear bio-polysaccharide produced by the alkaline deacetylation of chitin. Chitin is naturally found in the shell of shrimps and cramps. CH is non-toxic, biocompatible and biodegradable and has anti-microbial, anticancer, antiplaque, anti-tartar, and hemostatic effects. For the first time in 2007, the addition of CH to GICs was investigated and it was shown that the combination of 10% CH with GIC improves the GIC’s flexural strength and fluoride release (8). Besides, other studies have shown that adding CH to GICs led to an improvement of their compressive strength, wear resistance, and chemical properties (1,9).

A mechanical property of dental materials which may be related to the wear resistance of materials and their ability to remain sTable is surface hardness (10). Surface roughness plays an important role in the reflection of light in tooth-colored restorations and, thus, in the esthetics of such restorations. Rough surfaces lead to bacterial adhesion and plaque accumulation, and thereby increase the acidity and risk of decay which can endanger the longevity of restoration (2). The present study aimed to evaluate the effect of adding CH and micro-HP on microhardness and surface roughness of conventional and resin-modified glass ionomer cements which has not been studied previously.

## Material and Methods

-Mixing liquid of GIC with CH

Nearly 1.8 ml of glacial acetic acid was prepared up to 100 ml using distilled water in a 100 ml standard ﬂask to get 0.3 N acetic acid. Almost 20 mg of medium molecular weight CH (Sigma, Aldrich, USA) was weighed carefully using a weighing machine with the accuracy of ±0.0001g (A&D, GR+360, Tokyo, Japan) at room temperature and dissolved in 0.3 N acetic acid and prepared up to 100 ml using the same acetic acid in a 100 ml standard ﬂask to get 0.2 mg/ml CH solution. Then, 0.15 ml of 0.2 mg/ml CH solution was added to 0.85 ml of CGIC (Fuji II, GC, Tokyo, Japan) and RMGIC (Fuji II LC, Tokyo, Japan) liquids to form 15% v/v CH modified glass ionomer solution (11).

-Sample preparation

This experimental study was performed on 6 groups of 10 disk-shaped specimens. For the preparation of specimens, disc-shaped plastic molds with dimensions of 2 mm × 6 mm were used. The materials used in the present study are shown in Table 1.

**Table 1:**
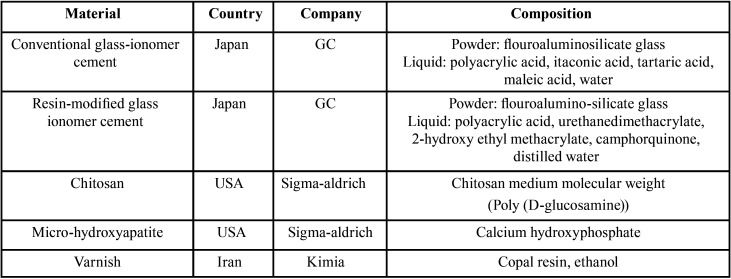
Materials used in the study.

Group I (CGIC control group): According to the manufacturer’s instructions, one scoop of powder was mixed with one drop of liquid for 25 seconds on a large surface of a cold glass slab using a plastic spatula. The upper and lower surfaces of the specimens were covered by a celluloid strip (Fintrec Transparent Matrix, M-TP, Pulpdent Corporation, Watertown, MA, USA). The discs were then placed between the two glass plates for 5.5 minutes until the GIC was completely set. A thin layer of copal varnish (Kimia, Iran) was applied on the surface of the samples.

Group II (RMGIC control group): One scoop of the RMGIC powder was mixed with two drops of liquid for 25 seconds, and the mixture was transferred into the molds. Then the upper surface of the specimens was cured for 20 seconds using a light-curing unit (Monitex, Bluelex, GT 1200, Taiwan) sticking to the glass slab with an intensity of 1200 mW/cm². A thin layer of copal varnish was applied on the surface of the samples.

Group III (CGIC + CH): The specimens contained CGIC powder and CGIC liquid mixed with CH solution. Powder and liquid were mixed in the same way as the first group. After the completion of the setting in 5.5 minutes, the mixture was removed from the mold and covered with varnish.

Group IV (CGIC + micro-HP): The specimens’ powder contained 90% wt CGIC, mixed with 10% wt micro-HP (Sigma, Aldrich, USA). The powders were weighed individually using a digital scale and mixed on glass plates using a plastic spatula. To prepare a homogeneous powder in all specimens, they were placed in cleaned amalgam capsules and mixed for 20 seconds using an amalgamator (Faghihi, FD + 300, Tehran, Iran). Then the powder and liquid were mixed according to the manufacturer’s instructions as mentioned in group I.

Group V (RMGIC + CH): The specimens contained RMGIC powder and RMGIC liquid containing CH solution. The mixture was prepared similar to group II.

**Figure 1:**
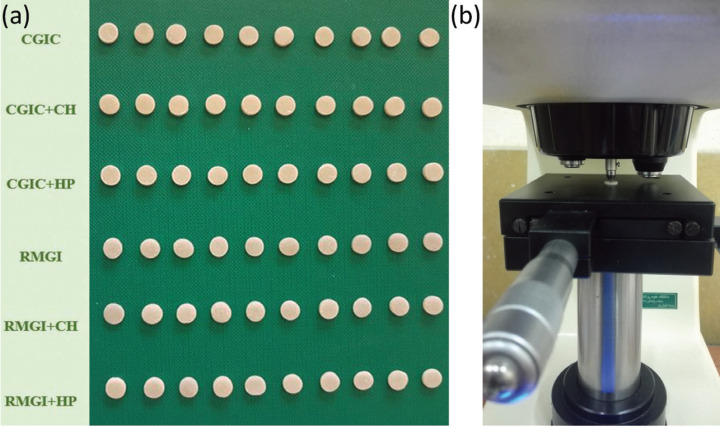
a) The specimens prepared in this study. b) Evaluating the microhardness of the experimental disc using a digital Vickers microhardness tester.

Group VI (RMGIC + micro-HP): The specimens’ powder contained 90% wt RMGIC and 10% wt micro-HP; the mixture was prepared similar to groups II and IV. The prepared specimens in this study have been shown in Figure 1a.

The samples were stored in distilled water at room temperature for 24 hours. Then the samples were polished with a low-speed handpiece (NSK, Japan) using polishing disks (Super Snap, Rainbow Technique kit, Shofu, Japan) with 4 different grits. The polishing procedure was performed for 30 seconds on both the upper and lower surfaces of each disk. To remove surface debris, the specimens were washed with distilled water for one minute in an ultrasonic bath (Renfert, GmbH, Germany). Consequently, the specimens were subjected to microhardness and surface roughness tests.

**Figure 2:**
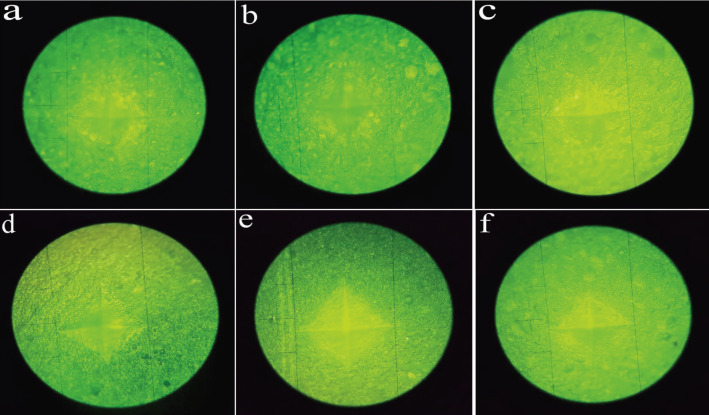
The indentation surface in the study groups (a: CGIC, b: CGIC+CH, c: CGIC+HP, d: RMGIC, e: RMGIC+CH, f: RMGIC+HP).

The hardness test was performed using a digital Vickers hardness testing device (SCTMC, 1000Z, China) with a force of 300 g / 15 seconds on each of the upper and lower surfaces. For each surface, 3 measurements were made, and the arithmetic mean values were recorded as the VHN levels of each surface (Fig. 1b). The distance between each of the indentation points was not less than one millimeter. The indentation surface can be seen in Figure 2.

Surface roughness (Ra µm) values of the upper surface of the specimens were measured using a contact profilometer (TESA Rugosurf, Switzerland). For each surface, 3 measurements were made, and the arithmetic mean values were used for statistical analysis. Data were recorded as the mean value and standard deviation of each group. The data were analyzed using SPSS version 16 (SPSS Inc., IL, US). Two-way ANOVA and Post-Hoc Tukey’s test were performed to show significant differences in subgroup comparisons. Paired T-Test was applied to compare the VHN values of two surfaces in each group (*P*<0.05).

## Results

The mean VHN values of the experimental groups are summarized in Table 2. Two-way ANOVA showed that the mean VHN values of the CH-containing groups were significantly higher than the micro-HP-containing groups (*P* <0.001). There was a statistically significant difference between the VHN values of the control groups of RMGIC and CGIC (*P* <0.001). However, no significant difference was found between the VHN of CGICs and RMGICs with the addition of micro-HP and CH (*P* = 0.703).

**Table 2:**
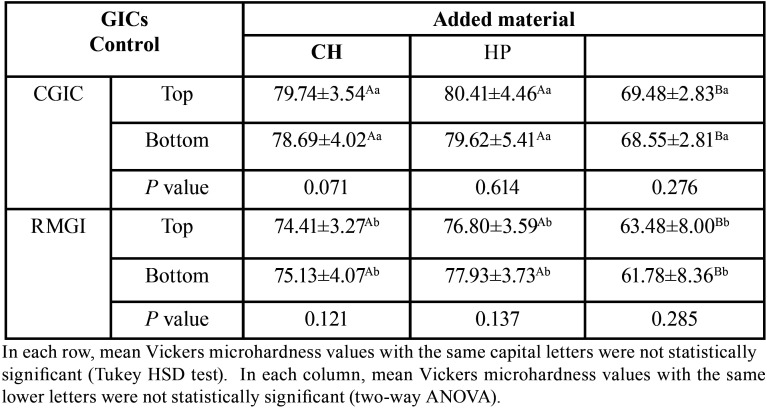
Mean Vickers microhardness values (VHN) ± standard deviations for the study groups.

**Figure 3:**
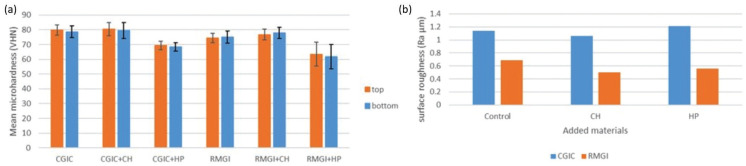
a) Means and standard deviations of Vickers microhardness values of the experimental groups; b) Means surface roughness values of the experimental groups.

Moreover, Post-Hoc Tukey’s test revealed that; the addition of micro-HP significantly reduced the VHN of both RMGIC and CGIC materials (*P* <0.001). However, the addition of CH did not induce a significant difference in the VHN of RMGICs and CGICs (*P* = 0.552). The top and bottom surfaces of the specimens also showed similar results in different groups. The mean VHN values and standard deviations in the top and bottom of each specimen in all groups can be seen in Figure 3 (a).

The mean and standard deviations of Ra values in each of the studied groups are summarized in Table 3. The mean Ra values were significantly lower in the CH- containing groups than the micro-HP-containing groups (*P* = 0.004). A statistically significant difference was found in the mean Ra values in the control groups of RMGIC and CGIC (*P* <0.001). No significant difference was found between the Ra values of CGICs and RMGICs with the addition of micro-HP and CH (*P* = 0.057).

**Table 3:**
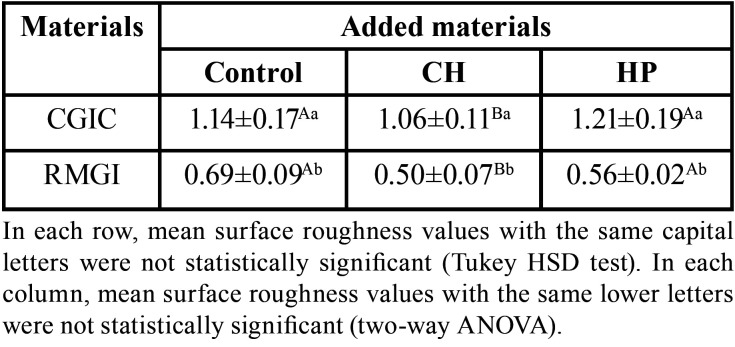
Mean surface roughness values (Ra/ µm) and standard deviations in the study groups.

Post-Hoc Tukey’s test revealed that; the addition of CH significantly reduced the Ra values in both RMGIC and CGIC materials (*P* = 0.004); There was no significant difference in Ra values of CGIC and RMGIC with the addition of HP (*P* = 0.700). The Ra values of micro-HP-containing groups were significantly different from CH-containing groups (*P* = 0.036). The mean surface roughness (Ra µm) values, Std. deviations in all groups can be seen in Figure 3 b.

## Discussion

In recent years, researches have been conducted on the incorporation of HP into glass ionomer cements to improve their bioactive properties and increase their mechanical strength (3,12). In a study by Moshaverinia *et al*. the compressive and flexural strength of nano-HP and fluoroapatite-containing glass ionomer cement were evaluated. The results of this study showed that the mechanical properties of the glass ionomer were improved by the addition of nano-HP and fluoroapatite (13). In the present study, micro-HP was used due to its similar hardness to natural teeth. Although the micro-HP nanoparticles are more similar to the mineral phase of the tooth in terms of particle size compared to micro-HP, there is a concern that nano-HP significantly increases the setting times in GICs (3). CH was also used in the present study. Due to the inter and intramolecular hydrogen bonding, CH has a rigid crystalline structure. It can also be used to reinforce the CGICs because of its biochemical properties (8). CH can also be used as a chemical or physical binder between glass filler and matrix in the GICs to improve their mechanical properties (9).

Surface hardness is one of the most important clinical properties of dental materials and can affect the clinical success of a restoration (14).

A previous study stated that adding 5 and 15 wt% micro-HP to GICs would reinforce the matrix, and result in better bonding between glass core and glass matrix and can increase GICs’ microhardness (3). In the present study, adding micro-HP to GICs resulted in a significantly decreased microhardness compared to the control groups and CH-containing groups, which is in line with the results of a previous study (15). In the present study, amalgamators were used for mixing the powders resulting in a non-uniform distribution of micro-HP or GIC powder where some parts of the surface contained only micro-HP or GIC powder. Furthermore, given that micro-HP has a lower density than GICs, an extra volume of micro-HP might alter the amount of fluid needed for the reaction of the powder and liquid in the cement (15). Moreover, HP can react with GIC as a non-reactive filler and a replacement of the reactive glass powder in an acid-base reaction (15). In the current study, the liquid was applied following the manufacturer’s instructions. Inadequate fluid in the mixture might likely alter the mechanical properties of the mixture; thus, it is assumed that this can be the cause of lower microhardness values observed in micro-HP-containing groups compared to the control and CH-containing groups. Moreover, the mechanical properties of the mixture can be influenced by the mixing time (16). In the present study, the mixing time was set similar to the manufacturer’s instructions. The addition of micro-HP to GICs might affect the mixing time which should be evaluated in future studies. This probable effect also might be a causative factor in the negative effects of micro-HP incorporation on the hardness of GICs.

CH is insoluble in water and organic solvents. However, it is dissolved in dilute aqueous acid solutions such as acetic acid (11). In the present study, CH was dissolved in an acetic acid solution mixed to 15% by volume with liquid GIC. The pH values of the modified liquids were maintained in an acidic range of about 1. The CH solution is a water-soluble cationic polyelectrolyte since the amine groups are protonated and charged positively at low pH. Due to the presence of an initial amino group with a value of pKa = 6.3, CH can be considered as a strong base. The presence of the amino group indicates that the pH changes the properties of the CH solution. The reaction between CH and GIC occurs through the NH2 group of CH and the functional group of the GIC (OH group and C = O group) (11). By increasing the concentration of CH, instead of reacting with the GIC components, CH molecules react with each other and become dislodged from the chain. In the present study, the addition of CH led to an increase in the hardness of both types of GICs although the increase was not significant. This might indicate that the amount of CH used in this study (15% of 0.2 mg/ml CH solution) does not interfere with the GIC setting reaction.

In the current study, the VHN values of CGICs were significantly higher than RMGICs. It seems that the resin matrix (2-hydroxyethyl methacrylate/ HEMA) might reduce GIC microhardness in two ways. The first mechanism is that the crosslink formed between carboxylate chains in GIC and HEMA molecules leads to the removal of polyacid chains from each other and prevents the formation of a crosslink that is normally formed through Ca2+. The second mechanism is that the water absorbed by the resin matrix (HEMA) can prevent the secondary curing reaction in the surface layer of the cement (17).

Other factors that affect microhardness include water-powder ratio, temperature, and humidity (18). To standardize these conditions, in the current study, all specimens were prepared in one day. The manufacturer’s instructions were followed in the mixing of the powder and liquid and the preparation of samples was done at room temperature. To prevent the early GIC water contamination and initial water absorption, a varnish layer was applied on both surfaces of each specimen. To standardize all groups, a transparent matrix tape was used on the surface of the specimens. Since the surface layer is in contact with the matrix tape, especially in RMGIC, it has a lower hardness than the other parts (19). To remove the surface layer with lower hardness and to simulate clinical conditions, in the present study, the specimens were polished before the hardness evaluation. This may lead to an increase in hardness, especially in samples containing RMGIC.

In a study by Cefley and Bayindir’s, the hardness values of the RMGIC were higher on the top surface of the specimens which has been attributed to the closer light source to the top. Moreover, light scattering and reduction in light intensity in the bottom were considered as the reasons for lower hardness in the bottom surface (20). Some other studies reported higher levels of hardness at the bottom of the samples (3,21). In the current study, the hardness values were similar in the top and bottom surfaces which was in line with the findings of a previous study (22). This could be attributed to the fact that in the RMGIC-containing specimens, the effective light transmission was carried out through the thickness of the discs, and sufficient energy for curing reached the bottom of the specimens. Moreover, in samples containing CGIC, the acid-base reaction was sufficiently carried out in the bulk of the specimens.

Surface roughness is one of the most important surface characteristics in the clinic (2). In this study, the profilometric analysis was used to measure Ra values because it is precise, adapTable, and easy to use. Ra is the algebraic mean height of the roughness component irregularities from the mean line measured in the sampling length.

 Investigating the CH + GIC matrix indicated that CH was completely mixed in the GIC matrix. CH has hydroxyl and acetamide groups that can form hydrogen bonds with powder’s hydroxyl groups and polyacrylic acid’s carboxylic acid group. This reaction can improve the GIC mechanical properties by reducing the interfacial tension between the GIC components (11). In this study, regardless of the GIC type, the CH-containing groups had the lowest Ra values, which could be due to the chemical bonding of CH with GICs leading to the formation of a homogeneous mixture. It has also been reported that the GICs liquid is also effective on surface roughness of GICs. To improve handling, efforts have been made to reduce liquid viscosity (2). In the present study, the viscosity of GICs Liquid was reduced by adding CH solution resulting in easier manipulation of powder and liquid. It seems that this will lead to the better mixing of powder and liquid in GICs and results in less void and lower surface roughness.

In some previous studies, the bond between HP and GICs has been reported to occur due to an interaction between carboxylates in polyacrylate polymers and Ca2+ in HP. Also, HP likely participates in the acid-base reaction of GICs by releasing ions. The apatite produced by HP and releasing ions from GICs can improve the mechanical properties of GICs (3). In the current study, the addition of HP did not significantly affect the mean Ra values of the specimens, while the GIC mixture with HP significantly reduced hardness. It seems that adding 10% micro-HP to the GICs powder did not cause an adverse effect on the surface roughness of GIC due to polishing.

The particle size in GICs is reported to affect surface roughness. The smaller the particle size is, the better the polishability of GICs will be (2,23). In a SEM evaluation, the surface of CGICs has shown higher voids than RMGICs (2). In the present study, all CGIC-containing groups had higher Ra values than the RMGIC-containing samples regardless of the type of substance added which was consistent with the results of a previous study (2). It seems that the smaller particle size in RMGIC is the reason for its smoother surface than the CGIC. Besides, it has been shown that the presence of resin improves the surface structure and better polishability of the surface resulting in reduced Ra values (2). It has been shown that the smoothest surface in GICs was obtained after the use of a Mylar Strip. However, a precise morphology of the restoration is rarely achieved through Mylar Strip alone and to form an anatomical contour, final finishing and polishing are required (24). In the present study, the specimens were polished to simulate clinical conditions. Fewer voids and better polishability of RMGIC-containing samples due to their smaller particle size and the presence of resin result in lower Ra values in comparison with those samples containing CGIC.

The present study has some limitations. The specimens were not evaluated under scanning electron microscopy (SEM) and confocal laser scanning microscopy (CLSM). Therefore, the effect of adding CH and micro-HP on the surface roughness and microhardness of RMGICs and CGICs should be evaluated using SEM and CLMS. Besides measuring the average roughness (Ra), other roughness parameters such as the peak height (Rp), depth of the lowest point of the profile (Rv), and total height of the roughness profile (Rt) also should be evaluated in future studies.

Based on the results obtained in the current study, CH and micro-HP modified GICs can be considered as promising restorative materials, and further studies are recommended on the effect of these two substances with different particle sizes on working time, setting time, bond strength properties, water sorption and solubility of GICs.

Within the limitations of this study, it can be concluded that:

1- Regardless of the type of GIC, the addition of CH to GICs reduced the surface roughness without posing a harmful effect on the surface hardness.

2- The addition of micro-HP to GICs reduced the hardness of both CGIC and RMGIC and did not significantly affect surface roughness of CGIC and RMGIC.

3- The CGIC investigated in this study showed higher hardness and surface roughness values than the RMGIC.
